# (*E*)-*N*′-(4-Bromo­benzyl­idene)-2-(8-quin­ol­yloxy)acetohydrazide mono­hydrate

**DOI:** 10.1107/S1600536809020418

**Published:** 2009-06-06

**Authors:** Jun Tan

**Affiliations:** a College of Biological and Chemical Engineering, Jiaxing University, Jiaxing 314001, People’s Republic of China

## Abstract

In the title compound, C_18_H_14_BrN_3_O_2_·H_2_O, the dihedral angle between the mean planes of the benzene ring and the quinoline ring system is 34.2 (3)°. In the crystal, the constituents are linked into chains by O—H⋯O, N—H⋯O and O—H⋯N hydrogen bonds.

## Related literature

For a related structure, see: Tan (2009 ▶). For background to the coordination chemistry of 8-hydroxy­quinoline and its derivatives, see: Chen & Shi (1998 ▶); Mona & Wageih (2002 ▶). For reference structural data, see: Allen *et al.* (1987 ▶).
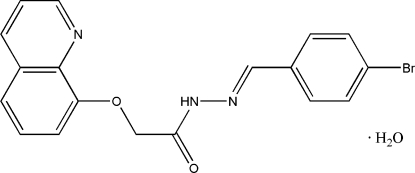


## Experimental

### 

#### Crystal data

C_18_H_14_BrN_3_O_2_·H_2_O
*M* *_r_* = 402.25
Monoclinic, 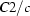

*a* = 21.95 (2) Å
*b* = 11.841 (8) Å
*c* = 13.057 (9) Å
β = 93.70 (2)°
*V* = 3387 (4) Å^3^
*Z* = 8
Mo *K*α radiation
μ = 2.45 mm^−1^
*T* = 295 K
0.20 × 0.18 × 0.15 mm

#### Data collection

Siemens SMART CCD area-detector diffractometer
Absorption correction: multi-scan (*SADABS*; Siemens, 1996 ▶) *T*_min_ = 0.640, *T*_max_ = 0.710
8378 measured reflections
2988 independent reflections
1334 reflections with *I* > 2σ(*I*)
*R*_int_ = 0.141

#### Refinement

*R*[*F* ^2^ > 2σ(*F* ^2^)] = 0.070
*wR*(*F* ^2^) = 0.178
*S* = 1.04
2988 reflections
227 parameters
H-atom parameters constrained
Δρ_max_ = 0.52 e Å^−3^
Δρ_min_ = −0.75 e Å^−3^

### 

Data collection: *SMART* (Siemens, 1996 ▶); cell refinement: *SAINT* (Siemens, 1996 ▶); data reduction: *SAINT*; program(s) used to solve structure: *SHELXS97* (Sheldrick, 2008 ▶); program(s) used to refine structure: *SHELXL97* (Sheldrick, 2008 ▶); molecular graphics: *SHELXTL* (Sheldrick, 2008 ▶); software used to prepare material for publication: *SHELXTL*.

## Supplementary Material

Crystal structure: contains datablocks global, I. DOI: 10.1107/S1600536809020418/hb2981sup1.cif

Structure factors: contains datablocks I. DOI: 10.1107/S1600536809020418/hb2981Isup2.hkl

Additional supplementary materials:  crystallographic information; 3D view; checkCIF report

## Figures and Tables

**Table 1:** Hydrogen-bond geometry (Å, °)

*D*—H⋯*A*	*D*—H	H⋯*A*	*D*⋯*A*	*D*—H⋯*A*
N2—H2⋯O3	0.86	2.04	2.860 (8)	160
O3—H19⋯N1	0.85	1.97	2.806 (8)	170
O3—H20⋯O2^i^	0.85	2.11	2.783 (7)	136

Symmetry code: (i) 
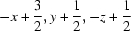
.

**Fig. 1.**
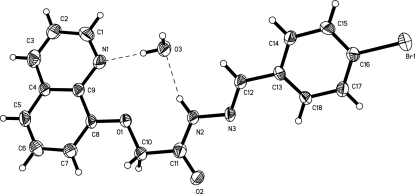
The molecular structure of (I), with displacement ellipsoids drawn at the 30% probability level. The dashed lines indicate hydrogen bonds.

**Fig. 2.**
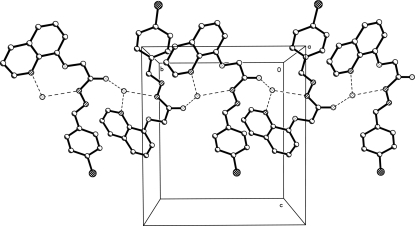
The structure of the chains formed via hydrogen bonds, H atoms have been omitted for clarity. The dashed lines indicate hydrogen bonds.

## supplementary crystallographic information

### Comment

8-Hydroxyquinoline and its derivatives constitute well known ligands in coordination chemistry (Chen & Shi, 1998; Mona & Wageih, 2002). In our search for new extractants of metal ions and biologically active materials, the title compound, (I), has been synthesized. We report here its crystal structure.

All bond lengths and angles are normal (Allen *et al.*, 1987), and are comparable to those in the related compound (*E*)-*N'*-[1-(4-Hydroxyphenyl)ethylidene]-2-(quinolin-8- yloxy)acetohydrazide methanol solvate (Tan, 2009). The mean planes of the benzene ring and the quinoline rings make a dihedral angle of 34.2 (3)°. In the crystal structure, the C_18_H_14_BrN_3_O_2_ molecules and the water molecules are linked into chains by O—H···O, N—H···O and O—H···N hydrogen bonds.

### Experimental

2-(quinolin-8-yloxy)acetohydrazide (2.18 g, 10 mmol), 4-bromobenzaldehyde (1.85 g, 10 mmol), ethanol (40 ml) and some drops of acetic acid were added to a 100 ml flask, and refluxed for 5 h. After cooling to room temperature, the mixture was filtered. Colourless blocks of (I) were obtained by slow evaporation of a acetone-methanol (1:2, *v*/*v*) solution over a period of 3 d.

### Refinement

The H atoms were initially located in a difference Fourier map, then relocated in idealised positions (C—H = 0.93–0.97Å O—H = 0.85 Å and N—H = 0.86 Å) and refined as riding with *U*_iso_(H) = 1.2*U*_eq_(C,*N*) or 1.5*U*_eq_(O).

### Crystal data

**Table:**

C_18_H_14_BrN_3_O_2_·H_2_O	*F*(000) = 1632
*M**_r_* = 402.25	*D*_x_ = 1.578 Mg m^−^^3^
Monoclinic, *C*2/*c*	Mo *K*α radiation, λ = 0.71073 Å
Hall symbol: -C 2yc	Cell parameters from 1037 reflections
*a* = 21.95 (2) Å	θ = 2.5–19.7°
*b* = 11.841 (8) Å	µ = 2.45 mm^−^^1^
*c* = 13.057 (9) Å	*T* = 295 K
β = 93.70 (2)°	Block, colourless
*V* = 3387 (4) Å^3^	0.20 × 0.18 × 0.15 mm
*Z* = 8	

### Data collection

**Table:**

Siemens SMART CCD area-detector diffractometer	2988 independent reflections
Radiation source: fine-focus sealed tube	1334 reflections with *I* > 2σ(*I*)
graphite	*R*_int_ = 0.141
ω scans	θ_max_ = 25.1°, θ_min_ = 2.0°
Absorption correction: multi-scan (*SADABS*; Siemens, 1996)	*h* = −15→26
*T*_min_ = 0.640, *T*_max_ = 0.710	*k* = −14→13
8378 measured reflections	*l* = −15→15

### Refinement

**Table:**

Refinement on *F*^2^	Secondary atom site location: difference Fourier map
Least-squares matrix: full	Hydrogen site location: inferred from neighbouring sites
*R*[*F*^2^ > 2σ(*F*^2^)] = 0.070	H-atom parameters constrained
*wR*(*F*^2^) = 0.178	*w* = 1/[σ^2^(*F*_o_^2^) + (0.0524*P*)^2^ + 0.013*P*] where *P* = (*F*_o_^2^ + 2*F*_c_^2^)/3
*S* = 1.04	(Δ/σ)_max_ < 0.001
2988 reflections	Δρ_max_ = 0.52 e Å^−^^3^
227 parameters	Δρ_min_ = −0.75 e Å^−^^3^
0 restraints	Extinction correction: *SHELXL97* (Sheldrick, 2008), Fc^*^=kFc[1+0.001xFc^2^λ^3^/sin(2θ)]^-1/4^
Primary atom site location: structure-invariant direct methods	Extinction coefficient: 0.0009 (2)

### Special details

**Table:**

Geometry. All e.s.d.'s (except the e.s.d. in the dihedral angle between two l.s. planes) are estimated using the full covariance matrix. The cell e.s.d.'s are taken into account individually in the estimation of e.s.d.'s in distances, angles and torsion angles; correlations between e.s.d.'s in cell parameters are only used when they are defined by crystal symmetry. An approximate (isotropic) treatment of cell e.s.d.'s is used for estimating e.s.d.'s involving l.s. planes.
Refinement. Refinement of *F*^2^ against ALL reflections. The weighted *R*-factor *wR* and goodness of fit *S* are based on *F*^2^, conventional *R*-factors *R* are based on *F*, with *F* set to zero for negative *F*^2^. The threshold expression of *F*^2^ > σ(*F*^2^) is used only for calculating *R*-factors(gt) *etc*. and is not relevant to the choice of reflections for refinement. *R*-factors based on *F*^2^ are statistically about twice as large as those based on *F*, and *R*- factors based on ALL data will be even larger.

### Fractional atomic coordinates and isotropic or equivalent isotropic displacement parameters (Å^2^)

**Table:**

	*x*	*y*	*z*	*U*_iso_*/*U*_eq_	
Br1	0.90959 (4)	−0.09454 (8)	−0.24581 (7)	0.0744 (4)	
O1	0.6370 (2)	0.0076 (4)	0.4059 (4)	0.0572 (15)	
O2	0.7206 (2)	−0.2340 (5)	0.3387 (4)	0.0720 (18)	
O3	0.6745 (2)	0.1677 (4)	0.2304 (4)	0.0709 (17)	
H19	0.6501	0.1798	0.2773	0.106*	
H20	0.7041	0.2133	0.2416	0.106*	
N1	0.5826 (3)	0.2041 (6)	0.3665 (5)	0.0569 (18)	
N2	0.7139 (2)	−0.0597 (5)	0.2679 (5)	0.0512 (17)	
H2	0.7015	0.0089	0.2724	0.061*	
N3	0.7481 (2)	−0.0944 (5)	0.1878 (4)	0.0498 (16)	
C1	0.5556 (4)	0.3013 (8)	0.3491 (7)	0.071 (2)	
H1	0.5638	0.3395	0.2893	0.085*	
C2	0.5152 (4)	0.3521 (7)	0.4142 (7)	0.071 (3)	
H2A	0.4977	0.4219	0.3980	0.085*	
C3	0.5024 (3)	0.2977 (8)	0.5002 (7)	0.069 (3)	
H3	0.4749	0.3292	0.5432	0.083*	
C4	0.5300 (3)	0.1940 (6)	0.5264 (6)	0.0469 (19)	
C5	0.5217 (4)	0.1349 (7)	0.6158 (6)	0.060 (2)	
H5	0.4951	0.1631	0.6621	0.072*	
C6	0.5512 (4)	0.0379 (8)	0.6369 (7)	0.067 (2)	
H6	0.5448	0.0005	0.6979	0.081*	
C7	0.5913 (3)	−0.0086 (7)	0.5699 (6)	0.060 (2)	
H7	0.6115	−0.0760	0.5860	0.072*	
C8	0.6007 (3)	0.0465 (6)	0.4799 (6)	0.0462 (19)	
C9	0.5708 (3)	0.1502 (6)	0.4560 (6)	0.0459 (19)	
C10	0.6614 (3)	−0.1020 (6)	0.4229 (6)	0.054 (2)	
H10A	0.6283	−0.1556	0.4271	0.065*	
H10B	0.6855	−0.1032	0.4879	0.065*	
C11	0.7011 (3)	−0.1375 (7)	0.3383 (6)	0.053 (2)	
C12	0.7666 (3)	−0.0121 (7)	0.1327 (6)	0.050 (2)	
H12	0.7585	0.0621	0.1509	0.060*	
C13	0.8002 (3)	−0.0349 (7)	0.0416 (5)	0.0441 (19)	
C14	0.8235 (3)	0.0555 (6)	−0.0099 (6)	0.051 (2)	
H14	0.8178	0.1285	0.0139	0.061*	
C15	0.8551 (3)	0.0384 (7)	−0.0965 (6)	0.058 (2)	
H15	0.8714	0.0993	−0.1304	0.069*	
C16	0.8623 (3)	−0.0703 (7)	−0.1320 (6)	0.050 (2)	
C17	0.8391 (3)	−0.1611 (7)	−0.0827 (6)	0.050 (2)	
H17	0.8437	−0.2340	−0.1073	0.060*	
C18	0.8085 (3)	−0.1412 (6)	0.0047 (5)	0.0459 (19)	
H18	0.7932	−0.2023	0.0397	0.055*	

### Atomic displacement parameters (Å^2^)

**Table:**

	*U*^11^	*U*^22^	*U*^33^	*U*^12^	*U*^13^	*U*^23^
Br1	0.0833 (7)	0.0776 (7)	0.0666 (6)	0.0062 (5)	0.0376 (5)	0.0007 (6)
O1	0.075 (4)	0.046 (3)	0.055 (3)	0.009 (3)	0.036 (3)	0.003 (3)
O2	0.096 (4)	0.037 (3)	0.089 (5)	0.008 (3)	0.056 (4)	0.004 (3)
O3	0.078 (4)	0.055 (4)	0.086 (4)	−0.008 (3)	0.050 (3)	−0.008 (3)
N1	0.060 (4)	0.048 (4)	0.066 (5)	0.004 (3)	0.025 (4)	−0.004 (4)
N2	0.053 (4)	0.041 (4)	0.063 (4)	0.001 (3)	0.033 (3)	−0.002 (3)
N3	0.053 (4)	0.045 (4)	0.055 (4)	−0.001 (3)	0.031 (3)	−0.007 (4)
C1	0.081 (6)	0.069 (7)	0.066 (6)	0.005 (5)	0.027 (5)	0.015 (5)
C2	0.076 (6)	0.059 (6)	0.080 (7)	0.027 (5)	0.023 (5)	0.004 (5)
C3	0.056 (6)	0.077 (7)	0.077 (7)	0.004 (5)	0.027 (5)	−0.014 (6)
C4	0.047 (5)	0.046 (5)	0.050 (5)	−0.001 (4)	0.024 (4)	−0.002 (4)
C5	0.069 (6)	0.064 (6)	0.051 (5)	−0.005 (5)	0.028 (4)	−0.011 (5)
C6	0.069 (6)	0.071 (7)	0.066 (6)	−0.004 (5)	0.037 (5)	0.007 (5)
C7	0.063 (5)	0.053 (5)	0.068 (6)	0.003 (4)	0.036 (5)	0.005 (5)
C8	0.053 (5)	0.044 (5)	0.045 (5)	−0.003 (4)	0.024 (4)	−0.004 (4)
C9	0.045 (5)	0.046 (5)	0.049 (5)	−0.003 (4)	0.016 (4)	−0.004 (4)
C10	0.064 (5)	0.044 (5)	0.058 (5)	−0.001 (4)	0.033 (4)	0.003 (4)
C11	0.053 (5)	0.046 (5)	0.062 (5)	−0.012 (4)	0.021 (4)	−0.008 (5)
C12	0.049 (5)	0.044 (5)	0.061 (5)	0.001 (4)	0.025 (4)	−0.004 (4)
C13	0.038 (4)	0.050 (5)	0.047 (5)	0.000 (4)	0.019 (4)	0.003 (4)
C14	0.052 (5)	0.040 (5)	0.063 (5)	0.002 (4)	0.023 (4)	−0.006 (4)
C15	0.055 (5)	0.049 (5)	0.072 (6)	0.000 (4)	0.027 (4)	0.015 (5)
C16	0.044 (4)	0.052 (6)	0.058 (5)	0.008 (4)	0.016 (4)	0.007 (4)
C17	0.054 (5)	0.049 (5)	0.047 (5)	0.003 (4)	0.013 (4)	−0.009 (4)
C18	0.053 (5)	0.041 (5)	0.045 (5)	−0.005 (4)	0.019 (4)	0.001 (4)

### Geometric parameters (Å, °)

**Table:**

Br1—C16	1.889 (7)	C5—H5	0.9300
O1—C8	1.371 (7)	C6—C7	1.394 (9)
O1—C10	1.417 (8)	C6—H6	0.9300
O2—C11	1.220 (8)	C7—C8	1.371 (10)
O3—H19	0.8500	C7—H7	0.9300
O3—H20	0.8500	C8—C9	1.417 (10)
N1—C1	1.309 (9)	C10—C11	1.509 (9)
N1—C9	1.370 (9)	C10—H10A	0.9700
N2—C11	1.344 (9)	C10—H10B	0.9700
N2—N3	1.389 (7)	C12—C13	1.464 (9)
N2—H2	0.8600	C12—H12	0.9300
N3—C12	1.292 (8)	C13—C18	1.364 (9)
C1—C2	1.402 (11)	C13—C14	1.381 (9)
C1—H1	0.9300	C14—C15	1.379 (9)
C2—C3	1.341 (11)	C14—H14	0.9300
C2—H2A	0.9300	C15—C16	1.380 (10)
C3—C4	1.402 (10)	C15—H15	0.9300
C3—H3	0.9300	C16—C17	1.368 (10)
C4—C5	1.383 (10)	C17—C18	1.380 (9)
C4—C9	1.422 (9)	C17—H17	0.9300
C5—C6	1.338 (10)	C18—H18	0.9300
			
C8—O1—C10	115.4 (5)	N1—C9—C4	122.9 (7)
H19—O3—H20	106.1	C8—C9—C4	118.3 (7)
C1—N1—C9	116.7 (7)	O1—C10—C11	111.8 (6)
C11—N2—N3	117.4 (6)	O1—C10—H10A	109.3
C11—N2—H2	121.3	C11—C10—H10A	109.3
N3—N2—H2	121.3	O1—C10—H10B	109.3
C12—N3—N2	113.6 (6)	C11—C10—H10B	109.3
N1—C1—C2	124.7 (8)	H10A—C10—H10B	107.9
N1—C1—H1	117.7	O2—C11—N2	123.8 (7)
C2—C1—H1	117.7	O2—C11—C10	118.4 (7)
C3—C2—C1	118.5 (8)	N2—C11—C10	117.8 (7)
C3—C2—H2A	120.8	N3—C12—C13	120.4 (7)
C1—C2—H2A	120.8	N3—C12—H12	119.8
C2—C3—C4	121.0 (8)	C13—C12—H12	119.8
C2—C3—H3	119.5	C18—C13—C14	118.7 (6)
C4—C3—H3	119.5	C18—C13—C12	122.9 (7)
C5—C4—C3	124.8 (7)	C14—C13—C12	118.3 (7)
C5—C4—C9	119.0 (7)	C15—C14—C13	120.5 (7)
C3—C4—C9	116.2 (7)	C15—C14—H14	119.7
C6—C5—C4	121.3 (7)	C13—C14—H14	119.7
C6—C5—H5	119.3	C14—C15—C16	119.1 (7)
C4—C5—H5	119.3	C14—C15—H15	120.4
C5—C6—C7	121.7 (8)	C16—C15—H15	120.4
C5—C6—H6	119.1	C17—C16—C15	121.4 (7)
C7—C6—H6	119.1	C17—C16—Br1	119.4 (6)
C8—C7—C6	119.1 (7)	C15—C16—Br1	119.1 (6)
C8—C7—H7	120.5	C16—C17—C18	118.1 (7)
C6—C7—H7	120.5	C16—C17—H17	121.0
C7—C8—O1	124.8 (7)	C18—C17—H17	121.0
C7—C8—C9	120.6 (7)	C13—C18—C17	122.2 (7)
O1—C8—C9	114.7 (6)	C13—C18—H18	118.9
N1—C9—C8	118.7 (6)	C17—C18—H18	118.9
			
C11—N2—N3—C12	−169.8 (7)	C3—C4—C9—N1	−0.8 (11)
C9—N1—C1—C2	−1.2 (13)	C5—C4—C9—C8	0.8 (11)
N1—C1—C2—C3	−0.6 (14)	C3—C4—C9—C8	178.9 (7)
C1—C2—C3—C4	1.8 (14)	C8—O1—C10—C11	−179.5 (6)
C2—C3—C4—C5	176.9 (8)	N3—N2—C11—O2	4.4 (12)
C2—C3—C4—C9	−1.1 (12)	N3—N2—C11—C10	−177.2 (6)
C3—C4—C5—C6	−177.9 (8)	O1—C10—C11—O2	−173.5 (7)
C9—C4—C5—C6	0.1 (12)	O1—C10—C11—N2	7.9 (10)
C4—C5—C6—C7	−0.4 (13)	N2—N3—C12—C13	−176.4 (6)
C5—C6—C7—C8	−0.1 (13)	N3—C12—C13—C18	6.5 (11)
C6—C7—C8—O1	−177.3 (7)	N3—C12—C13—C14	−174.6 (7)
C6—C7—C8—C9	1.0 (12)	C18—C13—C14—C15	−0.6 (11)
C10—O1—C8—C7	5.5 (11)	C12—C13—C14—C15	−179.6 (7)
C10—O1—C8—C9	−172.9 (6)	C13—C14—C15—C16	1.1 (12)
C1—N1—C9—C8	−177.8 (7)	C14—C15—C16—C17	−0.5 (11)
C1—N1—C9—C4	1.9 (11)	C14—C15—C16—Br1	−176.4 (6)
C7—C8—C9—N1	178.3 (7)	C15—C16—C17—C18	−0.6 (11)
O1—C8—C9—N1	−3.2 (10)	Br1—C16—C17—C18	175.3 (5)
C7—C8—C9—C4	−1.4 (11)	C14—C13—C18—C17	−0.6 (11)
O1—C8—C9—C4	177.1 (7)	C12—C13—C18—C17	178.4 (7)
C5—C4—C9—N1	−178.9 (7)	C16—C17—C18—C13	1.1 (11)

### Hydrogen-bond geometry (Å, °)

**Table:**

*D*—H···*A*	*D*—H	H···*A*	*D*···*A*	*D*—H···*A*
N2—H2···O3	0.86	2.04	2.860 (8)	160
O3—H19···N1	0.85	1.97	2.806 (8)	170
O3—H20···O2^i^	0.85	2.11	2.783 (7)	136

Symmetry codes: (i) −*x*+3/2, *y*+1/2, −*z*+1/2.
